# Supplementary material for: Splice-Junction-Based Mapping of Alternative Isoforms in the Human Proteome
Source: Cell Rep. Author manuscript; Available in PMC 2020 Jan 15. (PMC6961840; doi:10.1016/j.celrep.2019.11.026)
Supplement: 3 [file NIHMS1546469-supplement-3.zip › DF2/PXD006675/RightCoronaryArtery-134-P24043-NLRPEVNLK.pdf]

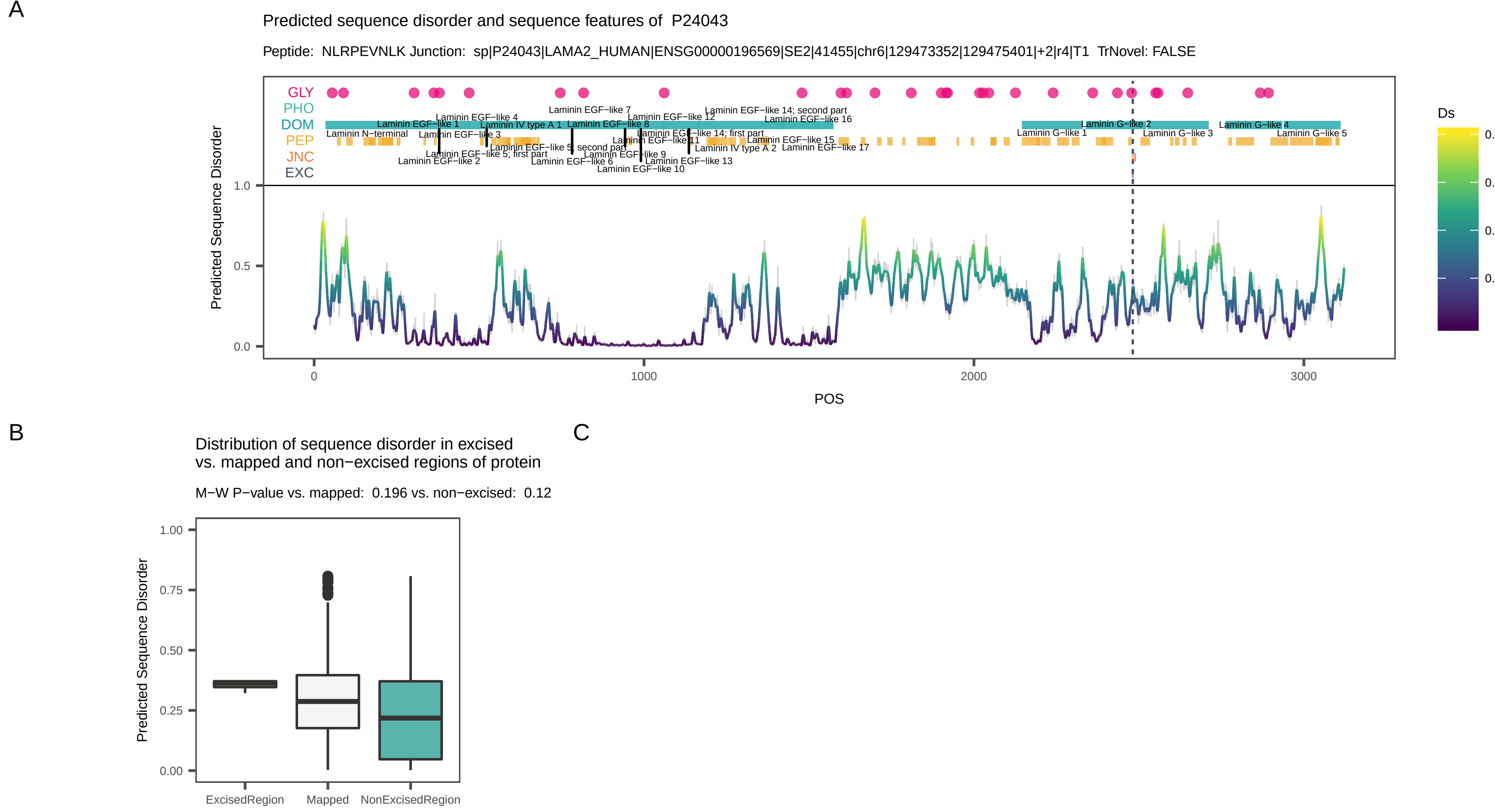
A
Predicted sequence disorder and sequence features of P24043
Peptide: NLRPEVNLK Junction: sp|P24043|LAMA2_HUMAN|ENSG00000196569|SE2|41455|chr6|129473352|129475401|+2|r4|T1 TrNovel: FALSE
GLY
PHO
DOM
PEP
JNC
EXC
Laminin N−terminal
Laminin EGF−like 1
Laminin EGF−like 2
Laminin EGF−like 3
Laminin EGF−like 4
Laminin IV type A 1
Laminin EGF−like 5; first part
Laminin EGF−like 5; second part
Laminin EGF−like 6
Laminin EGF−like 7
Laminin EGF−like 8
Laminin EGF−like 9
Laminin EGF−like 10
Laminin EGF−like 11
Laminin EGF−like 12
Laminin EGF−like 13
Laminin EGF−like 14; first part
Laminin EGF−like 14; second part
Laminin IV type A 2
Laminin EGF−like 15
Laminin EGF−like 16
Laminin EGF−like 17
Laminin G−like 1
Laminin G−like 2
Laminin G−like 3
Laminin G−like 4
Laminin G−like 5
Predicted Sequence Disorder
1.0
0.5
0.0
0
1000
2000
3000
POS
Ds
0.8
0.6
0.4
0.2
B
Distribution of sequence disorder in excised vs. mapped and non−excised regions of protein
M−W P−value vs. mapped: 0.196 vs. non−excised: 0.12
Predicted Sequence Disorder
1.00
0.75
0.50
0.25
0.00
ExcisedRegion
Mapped
NonExcisedRegion
C
